# Biopotential Signal Monitoring Systems in Rehabilitation: A Review

**DOI:** 10.3390/s21217172

**Published:** 2021-10-28

**Authors:** Arrigo Palumbo, Patrizia Vizza, Barbara Calabrese, Nicola Ielpo

**Affiliations:** 1 Department of Medical and Surgical Sciences, Magna Græcia University, 88100 Catanzaro, Italy; a.palumbo@dimes.unical.it (A.P.); calabreseb@unicz.it (B.C.); nicola.ielpo@unical.it (N.I.); 2 Mater Domini University Hospital, 88100 Catanzaro, Italy; 3 Interdepartmental Center of Services (CIS), Magna Græcia University, 88100 Catanzaro, Italy

**Keywords:** biomedical signal, monitoring system, rehabilitation, signal processing

## Abstract

Monitoring physical activity in medical and clinical rehabilitation, in sports environments or as a wellness indicator is helpful to measure, analyze and evaluate physiological parameters involving the correct subject’s movements. Thanks to integrated circuit (IC) technologies, wearable sensors and portable devices have expanded rapidly in monitoring physical activities in sports and tele-rehabilitation. Therefore, sensors and signal acquisition devices became essential in the tele-rehabilitation path to obtain accurate and reliable information by analyzing the acquired physiological signals. In this context, this paper provides a state-of-the-art review of the recent advances in electroencephalogram (EEG), electrocardiogram (ECG) and electromyogram (EMG) signal monitoring systems and sensors that are relevant to the field of tele-rehabilitation and health monitoring. Mostly, we focused our contribution in EMG signals to highlight its importance in rehabilitation context applications. This review focuses on analyzing the implementation of sensors and biomedical applications both in literature than in commerce. Moreover, a final review discussion about the analyzed solutions is also reported at the end of this paper to highlight the advantages of physiological monitoring systems in rehabilitation and individuate future advancements in this direction. The main contributions of this paper are (i) the presentation of interesting works in the biomedical area, mainly focusing on sensors and systems for physical rehabilitation and health monitoring between 2016 and up-to-date, and (ii) the indication of the main types of commercial sensors currently being used for biomedical applications.

## 1. Introduction

Biomedical wearable sensors allow the measurement of physiologic parameters in a continuous, real-time and non-invasive way, including a wide range of advances in electrocardiogram (ECG), electromyogram (EMG) and electroencephalogram (EEG)-based sensing platforms [1,2,3]. These platforms and their related sensors have different diagnostic and monitoring applications [4,5]. For example, physiological monitoring could support both diagnosis and ongoing treatment for many diseases involving movement disorders [6]. Furthermore, home-based motion sensing could assist the subject in rehabilitation path and falls prevention, helping him/her improve his/her independence and lifestyle [7]. Moreover, sensors acquire and analyze biomedical signals to monitoring the effectiveness of home-based rehabilitation therapies, for example, in stroke survivors, in patients undergoing surgery, in subjects involved in accidents or to evaluating the use of mobility assistive devices in older adults [8,9,10]. Moreover, the monitoring of physiological signals and parameters could also be a good support in many clinical and non-clinical applications, e.g., in sporting activities to evaluate performance and physical condition of athletes [1,11], in postural control to correct stability, or in a physiotherapy context after injury [12,13]. Generally, the measurement of physical activity parameters aids in guiding many types of applications; e.g., (i) monitoring physical activity during rehabilitation or in a physical therapy setting; (ii) evaluating the success of an intervention and tracking physical activity post-surgery; (iii) evaluating patient mobility; (iv) all (risk) detection; and (v) monitoring physical activity in patients with chronic diseases and disabilities involving movement disorders. Most recently, the coronavirus disease 2019 (COVID-19) pandemic has affected access to standard rehabilitation services, highlighting the need to define new rehabilitation perspectives as telemedicine [14,15]. During this period, the rehabilitation concept considerably changed: the need for home medical assistance for a new idea of rehabilitation phase in older people both with and without COVID-19, in patients affected by neuro-motor disease, in subjects with limited movements after injury or accident and athletes is becoming essential to help these people in maintaining their daily activities. In this context, tele-rehabilitation became an effective and well-accepted method of providing outpatient and community rehabilitation services to support family and caregivers in the assessment of the home environment, patient monitoring and outpatient therapies [16,17,18]. In emergencies such as the COVID 19 pandemic, access to health services is restricted due to the risk of infections and limitations of health resources [14,19]. For this reason, telemedicine services have proved extremely useful by providing home monitoring and rehabilitation solutions and thus minimizing the risk of infection. Survivors of COVID-19-associated pneumonia may experience a long-term reduction in functional capacity and muscle strength. Telerehabilitation (TR) programs could be effective for patients after COVID-19 [14]. However, few studies have assessed whether telerehabilitation for COVID-19 patients is an effective tool. In [20,21], telerehabilitation programs consist of home exercises for aerobic reconditioning, muscle strengthening, and healthy lifestyle education. The physiotherapist (PT) contacts the patient via video call via a dedicated platform to monitor progress. Moreover, physicians can add chest physiotherapy exercises for lung expansion and strengthening of the respiratory muscles. In these programs, a pulse oximeter as a monitoring device is also used.

Many healthcare devices for rehabilitation provide biosignals, such as blood pressure, blood glucose levels, EEGs, ECGs and EMGs [22]. The main bioelectrical signals are generated by the heart, the brain and the muscles, producing ECGs, EEGs and EMGs, respectively. ECG, EEG and EMG signals are characterized by low amplitude (generally, expressed in mV—millivolts) and low operating frequencies, from frequency Hz to some kHz range [23]. The acquisition, analysis and interpretation of these signals are fully reported in the literature [24,25,26,27,28,29,30,31]. Physical activity is often associated with the cardiovascular and muscular systems. Therefore, electrical signal variations cause ECG and EMG during athletic activities, and they are essential and commonly adopted parameters for healthcare management and rehabilitation protocols. In particular, EMG signal is the typical clinical recording method used to diagnose and monitor neuromuscular behaviours. Surface EMG (sEMG) allows extraction of information on muscle activation during a movement or effort, identifying impairment and functional alteration useful in clinical evaluation [32,33]. This information can be presented in different forms (e.g., amplitude, timing, morphology, muscle fibre conduction velocity or muscle coordination). They are relevant in many fields, from orthopaedics and neurorehabilitation to movement analysis in exercise and sport or aging [34,35]. This review aims to focus on EMG signal acquisition devices, also combined with other biosignals ECG and EEG, in rehabilitation pathways, especially for telemedicine applications. This contribution is proposed as a review by addressing questions such as (i) what are the most recent contributions in literature? (ii) what are the commonly used medical devices? (iii) how do these contributions and medical devices support physiological monitoring in rehabilitation? and (iv) what are the future directions and opportunities for EMG signal acquisition and analysis in a rehabiliation context? Many reviews are presented in the literature regarding biosignal acquisition devices for rehabilitation applications, but to the best of our knowledge, EMG signal has been considered only in specific context for single review. This review is thus a general but detailed comprehensive overview of EMG monitoring systems aiming to resume and to discuss the different and important solutions of EMG applications in different rehabilitation contexts. The key contributions of this review are outlined as follows. Section 2 presents a summary of basic concepts such as bioelectrical signalss and medical devices aiming to identify their main features and characteristics. These concepts are required background knowledge to this review. Section 3 reports the research methodology applied to define this review. Section 4 proposes an intense state-of-the-art about the last 5 years’ contributions in wearable sensors and platforms to summarize recent developments in the field of wearable sensors and systems used for rehabilitation. In Section 5, most commercial biomedical signal acquisition and processing sensors and devices have been reported comparing physical and performance characteristics. Section 6 has also been formulated to summarize and discuss the literature on EMG signal acquisition devices in rehabilitation and to explores their challenges and their future direction. Finally, Section 7 provides conclusions of the article.

## 2. Background

This section summarizes fundamental concepts needed to consider this review.

### 2.1. EMG and Rehabilitation

In the last decade, the new advanced devices and the increasing of computer science technologies have improved telemedicine applications [36]. A most important application regards telerehabilitation, which is still a new field in rapid growth. The advantage of telerehabilitation consists in the reduction of the costs both for health care providers and patients compared with traditional inpatient or person-to-person rehabilitation. Furthermore, telerehabilitation helps patients who live in remote places, allowing them to benefit from this technology. The primary application of telerehabilitation is regarding physiotherapy [37], and it is often associated with telemonitoring, referred to as the remote monitoring of physiological parameters, including ECG, blood pressure and oxygen saturation in patients with chronic diseases [38]. Physiotherapy applied to telerehabilitation is a valid approach both for musculoskeletal disorders and some physical diseases. Moreover, it contributes to improve patients’ posture and movement, supporting caregivers in creating a customized physical exercise program for physical rehabilitation [39,40,41]. In the rehabilitation field, the evaluation and the monitoring of the muscular conditions are needed to define a pathway aiming to develop and make stronger the correct voluntary muscle movements. The EMG signal is able to detect voluntary muscle activation giving information to encourage the correct patterns of activity. This EMG signal together with a robust, reliable and user-friendly acquisition and analysis system could be a strong support in rehabilitation cases to be used in assistive technology for helping people with severe disabilities [42]. The information about muscle activation can be expressed in different way, e.g., as amplitude, timing, morphology or spectral features, and it could be very useful and relevant in many fields ranging from orthopedics and neurorehabilitation to movement analysis in exercise and sport, from aging to obstetrics up to occupational and space medicine [33]. In the following sections, we report the potential clinical applications based on sEMG sensors in rehabilitation medicine with specific focus on (i) neurorehabilitation, (ii) stroke rehabilitation and (iii) sporting rehabilitation.

#### 2.1.1. EMG in Neurorehabilitation

Neurological rehabilitation medicine and its clinical research demonstrate that the damaged limb motor function can be restored to some extent through an efficient rehabilitation process. During this process, physicians must acquire and monitor the physical condition and physiological parameters to evaluate the training effect and, eventually, correct the follow-up rehabilitation training program. Information on muscle activation supports the physician in the clinical evaluation furnishing a reference for impairment and functional alterations. The ability of EMG signal in the measurement of this muscle activation makes this electrodiagnostic medicine technique very useful and relevant in neurorehabilitation, especially in the last four decades [43,44]. The application of sEMG techniques in neurorehabilitation is proposed in [34]. First, the authors report a review about the applications of EMG in neurological rehabilitation as support for the assessment and treatment of muscle spasticity and overactivity due to EMG’s ability to quantify alterations associated with these disorders. Moreover, the authors discuss their limited clinical applications. Another survey concerning the use of surface electromyography in neurorehabilitation is addressed by Manca et al. [45]. The authors collect information on (i) the current use of sEMG and its clinical potential, (ii) the professional figures primarily dealing with sEMG, (iii) the educational aspects and lastly, (iv) the possible reasons for its limited use in neurorehabilitation field. This survey research proposes and discusses different aspects of sEMG in neurorehabilitation ranging from current trends in its use; educational, technical and methodological features; and the translational outreach and potential utility of this technique for clinicians and patients.

The applications of surface EMG signals in neurorehabilitation regard: (i) the monitoring of neuromuscular pathologies, (ii) the prevention of work-related disorders and occupational therapy and (iii) the monitoring of neuromuscular changes and progress in severe patients. Information concerning the muscle activation during a movement or effort help physicians to evaluate and provide a clinical overview of both impairment and functional alteration. Another current common development of device in neurorehabilitation is represented by the acquisition of both EMG and EEG signals through biosignal amplifiers aiming to use physiological data to enhance their functionality in Brain Computer Interface (BCI) application. For example, the patient’s upcoming movements could be predicted by using electroencephalography (EEG) or electromyography (EMG). Always in BCI applications, EEG and EMG can be combined to either predict as many movements as possible or to enhance the reliability of movement prediction [46].

#### 2.1.2. EMG in Stroke Rehabilitation

Stroke represents one of the major causes of chronic motor disability among adults worldwide [47]. In addition, many stroke survivors suffer from hemiplegia, which makes walking difficult or even impossible. Therefore, rehabilitation represents an important treatment for the post-stroke patient to recover their muscle strength and motor coordination as well as to retrieve their nervous system [48]. Motor training and rehabilitation aim to be effective in enhancing muscle activity and improving neuromuscular control [49]. In this context, EMG-based methods could be a valid support in the detection of residual EMG activity and consequently in the control of exoskeletons in patients unable to generate sufficient joint torque, training them during the post-stroke period [50,51,52]. Electromyography, together with a controlled Neuromuscular Electrical Stimulation (NMES), generate the most benefits on motor recovery of upper limb function in clinical trials for patients with stroke [53]. In this direction, Monte-Silva et al. [54] propose a systematic review and meta-analysis concerning the effects of EMG-NMES on stroke upper limb recovery. Another significant contribution is furnished by Hameed et al. [55]. The authors discuss robotic devices as valuable tools to help patients with hand deficits in daily activities and restore hand functions by rehabilitation. Specifically, they highlight the potential of using sEMG in controlling hand robotic devices, including gloves and exoskeletons, for rehabilitation and assistance in daily activities. Furthermore, the authors in [56] investigate the possible use of EMG to detect hand/wrist extension movement intention to trigger robot-assisted training in individuals without residual movements. Specifically, they compared movement intention detection using an EMG detector with a sensorimotor rhythm-based EEG-BCI (Brain–Computer Interface) using only ipsilesional activity. The results show that EMG-based assisted therapy should be a valid and practical way to trigger robot-assisted training, furnishing also an easier interface and more compact dimensions than EEG-BCI devices.

#### 2.1.3. EMG in Sports Rehabilitation

Surface EMG can evaluate the status of skeletal muscles, assisting in muscular training and rehabilitation. Surface EMG is becoming a popular research tool in sport and rehabilitation sciences. In athletes’ rehabilitation, EMG is useful to analyze muscle dysfunction, to detect not-correct muscle activation patterns and to assist in establishing and assessing treatment outcomes [57,58,59]. The correct utilization of muscles and the rapid identification of anomalous muscle pattern activation help the athletes to improve their activities and to prevent the risk of injury [60]. The dynamic analysis of muscles performed by sEMG is particularly interesting in sport, especially in injury prevention. For example, the analysis of the sMEG signal could improve the performance of a task by evaluating the muscular activation and/or muscular fatigue [61]. An important application of EMG in sporting rehabilitation regards the fatigue analysis in triceps brachii. Hussain et al. [62] report an interesting review summarizing and analyzing the research findings regarding analysis of fatigue in the human triceps brachii (TB) muscle through surface electromyography (sEMG) observations. Other applications are in postoperative rehabilitation following rotator cuff repair, as reported in [63], or in monitoring different responses of skeletal muscles subjected to external stimuli such as hypoxia and physical activity [64]. Wearable biosensors are becoming most important in real-time physiological monitoring useful in athletic performance analysis, injury and recovery time assessment, thus supporting athletes, trainers and coaches in characterizing the daily demands of sports [65]. A sports rehabilitation monitoring system based on wearable sensors and Internet of Things technology has been developed in [66]. This system includes sensors to acquire and monitor ECG signals, EMG signals, motion posture, body temperature and other physiological parameters. The experimental results show that the system can closely monitor changes in vital signs while providing real-time monitoring and feedback. Furthermore, the acquired physiological data can be analyzed to support physicians in formulating effective rehabilitation training programs. Another contribution in the wearable system for EMG acquisition and analysis to evaluate athlete performance is proposed in [67]. The authors investigate the validity and reliability of their proposed sEMG system to characterize muscle activation patterns during isokinetic knee extension and flexion.

### 2.2. EMG Signal Acquisition: General Considerations

The main bioelectrical signals are generated by the heart, the brain and the muscles, producing ECGs, EEGs and EMGs, respectively. The acquisition, analysis and interpretation of these signals are fully reported in literature [24,25,26,27,28]. Electrical signal variations generate EMG signals during muscular activities [68]. Therefore, in physical activity monitoring and the evaluation of muscle conditions, EMG has become an important and commonly adopted parameter for healthcare management and rehabilitation protocols. High-quality recording of EMG signal is performed by well-designed instrumentation, and it is important to guarantee a correct processing and feature extraction [69].

#### 2.2.1. EMG Signal Features

EMG is an electrodiagnostic medicine technique to evaluate and record the muscular electrical signal generated by skeletal muscle activities [70]. The EMG signal measures electrical currents generated in muscles during their contraction, representing neuromuscular activities [71]. Thus, EMG provides excellent information about the health of muscles and the function of motor neurons, which transmit electrical signals to muscle cells allowing their contraction. Generally, EMG signals are characterized by a frequency range from 20 up to 2000 Hz and by amplitude from about 50 μV to 20 mV [72]. The features analysis of EMG signal could offer body muscle activity information, such as fitness, fatigue and endurance level and gesture, such that the biomechanics of human medical abnormalities or activity levels can be detected and analyzed. The evaluation of muscle activation during a movement or effort provides useful information on impairment and functional alteration. It is widely used in medical rehabilitation, human–machine interface, biomedical research and other fields. The current applications of EMG mostly regard physiological investigations, monitoring of neurological disorders, planning of treatments, assessment of interventions and control of prostheses and robots [73]. Electromyography can be performed using two electrodes: surface (or skin) electrodes or inserted (or wire and needle) electrodes. Surface electrodes are used to monitor the overall activity of a muscle, while the second type is generally used to reveal the electrical activity of a nerve [12]. EMG signals represent the state of limb muscle activity, reflecting the movement of skeletal muscle and the command information of the nervous system, which are very useful in stroke rehabilitation treatment. The acquisition and the analysis of EMG signals are useful to classify and recognize different limb movements, supporting the identification and the study of limb movements and their characteristics. Specifically, sEMG provides a non-invasive and global measurement of muscle activity, and it may be suitable for applications in movement analysis requiring frequent assessments or information on the patterns of activation of multiple muscles [74]. For example, surface EMG could be a valuable tool in sport, rehabilitation and clinical assessment to quantitatively measure progress and evaluate treatment outcomes.

#### 2.2.2. EMG Instrumentation Characteristics

**Figure 1:**
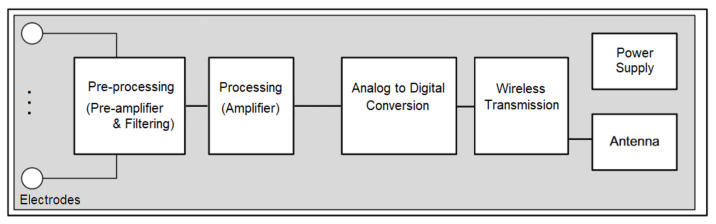
General EMG acquisition system.

The EMG signal is a complex and non-stationary physiological signal characterized by a low amplitude and low frequency values. Therefore, its acquisition is not easy to perform due to noise. The noise includes three main components: (i) the noise of the electronic acquisition equipment, (ii) the noise generated by skin-electrode contact and (iii) the noise added by the power frequency interference. Therefore, a well-specified acquisition and analysis system must be designed to improve the quality of EMG signal acquisition and its spatial and temporal resolution. Generally, an EMG acquisition system is composed of: (i) electrode, (ii) preprocessing stage (pre-amplifier and filtering), (iii) processing stage (amplifier), (iv) analog to digital conversion, (v) power supply and (vi) wireless transmission module [75], as shown in Figure 1.

Synthetically, the amplifier magnifies the difference in voltage between the inputs, attenuating the unwanted noise, aided by analog filters; then, the amplified signal is measured using an analog-to-digital converter (ADC), and this digitized signal allows further computerized analysis. Moreover, the signal acquisition can be performed into two different modalities: the monopolar acquisition performs the difference between a signal detected on the electrode concerning a remote reference placed in an electrically inactive area; the bipolar acquisition, instead, performs the difference between the signal detected on the electrode concerning another electrically active electrode. Finally, the wireless transmission module aims to connect the system with an external PC for EMG data analysis and processing as well as display, control, storage and query functions. This module is also the key element to achieve a portable performance of rehabilitation training. As reported above, EMG signal is affected by the environment, physiological and equipment noise components, so the acquired EMG signal contains a lot of information, useful and not. Therefore, a pre-processing module is necessary to remove baseline drift and power frequency by using a filtering stage composed of a low-pass filter and a high-pass filter [70]. The denoising and the removal of baseline wander are useful to reject the useless frequency signals cleaning the EMG signal by unwanted components. Moreover, a 50/60 Hz filtering (known as notch filter) eliminates 50/60 Hz power line frequency and harmonic noise in EMG. In particular, high-pass filters attenuate low-frequency components in the signal, and its low-frequency cutoff must be accurately chosen because it could cause initial amplitude loss of slowly changing signals, waveform distortion, decreasing the latency to the peak of the waveform and introducing artefacts [69]. On the other hand, low-pass filters attenuate high frequencies, and in this case, its high-frequency cutoff must also be opportunely chosen due to its influence in the reduction of the amplitude and rise time. EMG signal in output from the conditioning stage (preprocessing and processing modules) should be sent to an acquisition device for data recording, analysis and/or storage. This system is generally composed of an Analog-to-Digital Converter (ADC) that discretizes the signal in both time and amplitude, assigning a digital value to the amplitude at defined time points. This procedure is needed to further perform signal analysis for both clinical and research diagnostic purposes. Generally, the EMG front-end system should be satisfied well-defined specifications. The main specifications regard [76]:

- Accuracy: this characteristic is related to the implementation of the differential amplifier, ADC and several other components connected to inherent noise; the aim is to optimize each used component to minimize noise, ensuring accuracy;
- Sensitivity: this features on the ADC resolution and consequently the overall resolution of the system; it allows the physicians to understand the limits of their reading;
- CMRR: this is the Common-Mode Rejection Ratio, and it expresses the ability of the differential amplifier to reject common-mode signals; it plays a crucial role in avoiding 50–60 Hz power line interference;
- Input impedance: the optimization of this value is relevant in differential amplifier selections and implementations related to different user skin types and electrode interfaces;
- Input range: this specification regards hardware implementation and ADC, specifying the range of the biosignal that can be picked up without saturating the amplifier. A larger input range is preferred to acquire the entire signal, but this requires an expansion of signal resolution;
- SNR: this is the Signal-to-Noise Ratio, and it is the ratio between the signal’s amplitude and the background noise.

## 3. Research Methodology

This review has been conducted following the Preferred Reporting Items for Systematic Reviews and MetaAnalyses (PRISMA) item [77]. This research aims to investigate and provide a review of existing research on biosignal monitoring systems in the rehabiliation field. Scientific contributions and commercial devices have been chosen based on their contents and applications, closely related to the objective of this paper.

For scientific papers, the chosen databases were PubMed, MDPI, Springer, ACM Digital Library and Science Direct, as reported in Table 1.

The main research questions (RQ) of this study are:

- RQ1: what are the most recent contributions in literature?
- RQ2: what are the commonly used medical devices?
- RQ3: how do these contributions and medical devices support physiological monitoring in rehabilitation?
- RQ4: what are the future directions and opportunities for EMG signal acquisition and analysis in a rehabiliation context?

**Figure 2:**
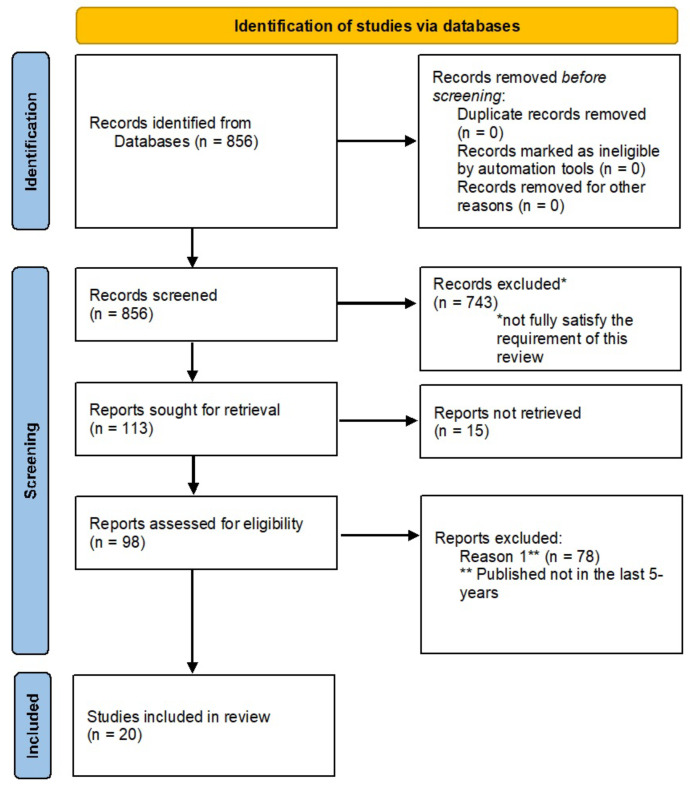
Flow diagram about research methodology.

Based on these research questions, while reviewing the existing research on keywords *Biosignals*, *Acquisition device*, *Wearable device*, *Monitoring*, *Rehabilitation* and *Telemedicine*, a total of 856 were identified as interesting for the topic of this review from 2004 to the present. From these resulting articles, 743 were removed because they did not fully satisfy the requirements of this review. Starting from these 113 remain papers, 15 contributions were also excluded because they did not report explicit considerations about EMG signals. Finally, from 98 studies, 20 contributions about wearable monitoring systems were chosen among papers published in the literature in the last 5 years to be analyzed in depth. A flow diagram illustrating our review methodology process is shown in Figure 2.

## 4. Wearable Devices for Rehabilitation

Rehabilitation consists of an iterative process involving assessments and specialized training, which unfortunately are often limited by healthcare centres’ restricted resources. To overcome this limitation, wearable technology should be an important, potential and valid solution to objectively assess and monitor patients inside and/or outside clinical environments. The information extracted by the use of this technology should provide a more detailed evaluation of the impairment, also allowing the identification of rehabilitation therapies [78]. The advantage of wearable devices in terms of portability, low cost and unobtrusive sensors makes this technology highly efficient in tracking movements aiming to enhance patient care with neurologic or musculoskeletal conditions. Furthermore, these sensors enable quantification of motor behaviour useful in compensation motor recovery mechanisms, remote monitoring, telerehabilitation and robotics [79,80,81].

**Figure 3:**
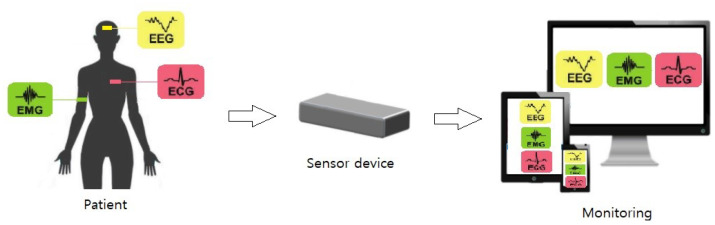
General setup of biosignal monitoring system.

Electrical biosignals are important indicators of the health and fitness condition of the human body. The acquisition and analysis of biosignals such as ECG, EMG and EEG through real-time e-health monitoring systems allow the extraction of relevant and useful information to achieve better healthcare in terms of observation, diagnosis and treatment [82]. A general setup of these systems is reported in Figure 3:

EEG, ECG and EMG signals are extracted by electrodes placed on the patient and acquired by sensor devices able to process these signals and transmit them on accurate instrumentation for monitoring.

Generally, these systems present heavy drawbacks regarding the limitation in acquiring and sending data at high rates, the low energy efficiency and the restricted portability due to their large size and weight. To overcome these limitations and make these systems more efficient, wearable devices are becoming essential in daily and clinical practice to allow continuous monitoring of human activity in terms of changes in biological signals. The increasing trends of wearable devices and the multimodal acquisition of different biosignals are crucial for advancing disease-diagnosis and treatment. Wearable devices perform activity monitoring through two main processes: (i) data acquisition and preprocessing; (ii) transmission, analysis and classification of acquired data. Signal preprocessing, for example, includes amplification and filtering stage; signal analysis, instead, involves averaging or extraction of relevant features to be used as training data for classifier [83].

In literature, many contributions are available concerning the design and the implementation of wearable sensors aiming to define platforms of multimodal acquisition and recognition of different biosignals, such as electroencephalography, electromyography and electrocardiography, for continuous and automatic monitoring of human health status, improving diagnosis, follow-up and therapeutic strategies of several disorders. Wearable devices usually involve smart sensors to detect and monitor a set of physiological parameters aiming to support their continuous monitoring for diagnostic, therapeutic and control purposes [84]. The great demand of the aging population for healthcare management needs the use of these wearable medical devices to monitor personal health information in real-time to prevent diseases and emergency health risks. Today, many wearable healthcare devices provide biosignals, such as EEGs, ECGs, EMGs, blood pressure or blood glucose levels. Electrical signal variations cause ECG and EMG during muscular activities, and they are important and commonly adopted parameters for healthcare management and rehabilitation protocols.

Zhao et al. [84] propose a wearable monitoring device for upper limb rehabilitation. This device integrates ECG/EMG sensors with data acquisition boards to obtain accurate signals during robotic glove assisting training. The ECG/EMG signals are acquired, preprocessed, digitized and transmitted to a remote receiver via a low-energy Bluetooth module. In addition, a software platform was developed for data analysis by integrating different algorithms to visualize ECG/EMG information and extract patterns of interest. EMG and ECG sensors monitor the hand activities and the relative changes in the physiological status of a subject, respectively. The results show that monitoring ECG and EMG signals assist the subject in improving upper limb rehabilitation according to specific treatment conditions and the users’ demands.

Liu et al. in [85] propose a portable and wireless acquisition system to acquire physiological signals. The system mainly consists of a portable device, a graphic user interface (GUI) and an application program for displaying the signals on a computer or a smart device. This device is characterized by eight measuring channels, a powerful microcontroller unit, a lithium battery, Bluetooth 3.0 data transmission and a built-in 2 GB flash memory. The results show that as this system can measure signals in real-time, supporting physicians and researchers can perform experiments collecting physiological signals of interest.

Park et al. [86] report about an energy-efficient integrated circuit architecture of a 128-channel Δ-modulated ΔΣ analog front-end (Δ-ΔΣ AFE) for 1024-channel neural recording microsystems. The proposed platform is based on the modularity of 128 channels and consists of eight multi-shank neural probes connected to individual AFEs. In addition, a spectrum equalization scheme has been implemented to reduce area and energy consumption, taking advantage of the inherent spectral characteristics of neural signals (most of the energy is present in low frequencies). The following features characterize the designed Δ-ΔΣ AFE: each single-channel AFE consumes 3.05 μW from 0.5 and 1.0 V supplies in an area of 0.05 mm^2^ with 63.8-dB signal-to-noise-and-distortion ratio and 3.02 noise efficiency factor.

An analog front-end AFE with two-channel acquisition is described in [87]. It is characterized by high impedance for low power application of bioelectrical activity. The proposed architecture comprises a programmable gain amplifier (PGA) and a 10-bitΣΔ (SDM-ADC). The overall gain is programmed through the flip-over-capacitor feedback and proposed reconfiguring in the PGA. The AFE measured frequency response from 50 Hz to 360 Hz with an SNR of 63 dB, power consumption of 11 mW, programmable gains from 52.6 dB to 72 dB and an input-referred noise of 3.5 µV in the amplifier bandwidth.

In [88], a multi-channel data acquisition system to record bio-electrical signals is proposed. The system consists of eight front-end acquisition modules and a synchronization module useful for reliable synchronization of all acquired signals. Each front-end acquisition module uses a separated universal serial bus data link to the computer. It is synchronized with other modules by an external clock, providing the time-base for the microcontrollers. The generated synchronization error is smaller than 10 μs, so the system is suitable for real-time analysis of movements. Furthermore, each analog front-end circuit is based on the highly integrated chip ADS1299, which contains analog filters and simultaneous digitalization of eight bipolar channels. Therefore, the proposed system can support real-time recordings of up to 64 bipolar channels. Lastly, raw data are analyzed and stored on a personal computer or a single-board computer.

A most recent contribution is given by Tran et al. in [89]. The authors present a four-channel, power-efficient and low-noise neural recording analog front-end (AFE) integrated circuit (IC). The overall architecture is composed of a four-channel neural recording analog front-end. Each front-end channel consists of a low-noise amplifier (LNA), a programmable gain amplifier (PGA) and buffers. The four-channel AFE is followed by a 4-to-1 multiplexer (MUX) and the analog-to-digital converter (ADC). The overall system presents a programmable gain range from 45 dB to 63 dB, and it achieves integrated input-referred noise of 3.16 μVRMS within the 10 kHz bandwidth, a noise efficiency factor of 2.04, a power efficiency factor of 4.16 and =power consumption of 2.82 μW per channel powered from the 1-V supply voltage.

A modular and wearable system for the acquisition and wireless transmission of biological signals is proposed in [90]. This system has been configured for different signals, such as ECG and EMG signals, and it is based on the ADS1294 Medical Analog Front End and the CC3200 microcontroller, both from Texas Instruments. It is a portable solution supplied by two Li-ion charged batteries. The results are promising in size, physical reduction, robustness in the wireless transmission and reliability in data acquisition and processing.

Another contribution is proposed in [91] by Sarker et al. A compact and wearable portable bio-signal acquisition device has been designed and implemented. It is characterized by real-time data wireless transmission and low energy consumption. The system has been defined to acquire ECG and EMG signals at eight channels with a 24-bit resolution/channel configuration and 500 samples/s. Moreover, the device has been used in an IoT-based system as an example of possible integration.

Mazzetta et al. [92] propose a stand-alone wearable sEMG system for monitoring muscle activity in real time. This system can detect the muscle activation potentials, and it embeds the complete real-time data processing thanks to an integrated low-power microcontroller. The system is optimized for power consumption, compactness and energy autonomy, so it can be used for valuable diagnostic data sets for patients during their day-to-day life. Moreover, the results in testing the system report an achieved specificity and sensitivity in recognizing exact activity timing over 87% and 82%, respectively, with the advantage of being wireless and comfortably wearable.

The system presented in [93] consists of a non-contact ECG sensor with a fully integrated analog front-end (AFE), a temperature sensor, an accelerometer and a Bluetooth Low Energy (BLE) module for multiparameter real-time monitoring. Small dimensions characterize it, and it can be used by inpatient, outpatient, people with disabilities or aging people who live alone. Data processing is performed by an Android application, sending alerts to authorities in case of an emergency.

Kim et al. [94] present a low-power, multimodal analog front-end (AFE) for wearable health monitoring sensors. It is based on novel system architecture and very large scale integrated circuit design methods with CMOS technology. Three sensors for bio-potential, photoplethysmography (PPG) and bioelectrical impedance analyzer (BIA) are integrated for low dimension and power consumption. Results showed high-quality AFE permitting users to effortlessly self-monitor multiple clinically relevant physiological parameters.

Authors in [95] proposes a novel analog front-end (AFE) to investigate three features: (i) voltage-dependent input impedance, (ii) bandpass amplification and (iii) stray capacitance reduction by using capacitive electrocardiogram (cECG) or capacitive electromyogram (cEMG) measurements in seven human subjects. Performance evaluation indicates that the proposed AFE can provide a feasible balance between sensitivity and stability in capacitive biopotential measurements (CBMs). Thus, it could be a versatile replacement for the conventional voltage follower used in CBMs.

Biagetti et al. [96] present a low-cost wearable wireless system for the acquisition of surface electromyography (sEMG) and accelerometer signals aiming to monitor human activity when performing sport and fitness activities, as well as in healthcare applications. The proposed system consists of several ultralight wireless sensing nodes that can acquire, process and efficiently transmit the motion-related (biological and accelerometer) body signals to one or more base stations through a 2.4 GHz radio link using an ad hoc communication protocol designed on top of the IEEE 802.15.4 physical layer. In addition, a user interface software for viewing, recording and analyzing the data was implemented on a controlled personal computer connected through a USB link to the base stations. To demonstrate the system’s capability to detect the user’s activity, data recorded from a few subjects were used to train and test an automatic classifier to recognize the type of exercise being performed. The system was tested on four different exercises performed by three people; the automatic classifier achieved an overall accuracy of 85.7% combining the features extracted from acceleration and sEMG signals.

Another contribution proposed by Biagetti et al. [97], following the previously developed system, regards the design of a wireless sensor device for the real-time acquisition of bioelectrical signals, such as EMG and ECG. This device aims to furnish a complete stream of data suitable for human activity detection, motion analysis and technology-assistance for people with physical or cognitive impairments. Six electrodes are considered to allow up to three independent bioelectrical channels, each with 24 bits of resolution and a sampling rate up to 3.2 kHz. Moreover, a Bluetooth Low Energy wireless link has been chosen to interact with many consumer electronics devices. Specifically, this contribution investigates data rate restrictions imposed by these devices proposing a strategy aiming to maximize the available bandwidth and reliability of the transmission.

Xian Li and Ye Sun [98] present a button-like wearable wireless non-contact system for long-term multiple biopotential signal (ECG, EMG and EEG) monitoring. This system is based on an ultra-high input impedance of the analog front-end for non-skin contact detection. The system is powered by a 150 mAh rechargeable Li-ion battery and packaged into a 39 mm × 32 mm × 17 mm 3D printed small box for a total weight of 24.0 g. A power management circuit is included to provide a dual power supply for operational amplifiers. The system’s performance has been evaluated through multiple motion scenarios with different types of cloth, and the results show the feasibility of long-term biopotential monitoring for daily application without affecting daily activities.

In [99], the authors present a single-channel amplifier to simultaneously acquire the ECG signal and the impedance respiration signal. The system is based on the oversampling and fast digital lock-in technology. It uses capacitive reactance of a capacitor changing with the signal’s frequency to satisfy the different impedance requirements of both the respiratory impedance signal and ECG signal. The preprocessing stage has been designed to improve the common-mode rejection ratio (CMRR) and the signal-to-noise ratio (SNR). ADS1294R (four-channel 24-bit ADC with integrated respiration impedance and ECG front-end) has been included to detect the ECG signal, and respiration signal and STM32F103RET6 has been adopted for signal processing. The results show as the designed circuit can support the simultaneous acquisition of multiple human physiological signals in a signal channel. Moreover, it can also detect other impedance variation signals and bioelectrical signals such as EMG, EOG and EEG signals.

The authors of [100] propose their contribution in the design of a portable device for ECG, EMG, EEG and Electrooculogram (EOG) signal monitoring aiming to support diagnosis and the evolution of several diseases. The processor satisfies the suppressing of baselines wander (0.1–0.5 Hz) and power line interference noise (50/60 Hz), and it provides to switch between low noise–high CMRR mode and average noise-average CMRR mode. Moreover, a bandpass and a band-stop FIR filter have been developed. The processor also contains a Successive Approximation Register (SAR) DAC for the controlling signal. It is designed in Spartan-3E FPGA and 0.18 μm CMOS TSMC technology for a total of 33,005 μm^2^ area and power consumption of 0.382 mW.

Lee et al. [101] present a novel wireless ExG sensor tag with a multi-channel physiological signal acquisition (PSA) system aiming to acquire biopotential signals, such as ECG, EOG and EMG. Furthermore, a mixed-signal processor system-on-chip (SoC) and Bluetooth Low Energy (BLE) chip have been implemented for real-time recording and wireless transmission, respectively. This system is optimized for power efficiency, and it can be easily achieved in 12 h with a 200 mAH battery of continuous recording of ExG signals in healthcare applications.

Flexible architecture of a multi-purpose physiological signal (e.g., ECG, EMG) recorder is presented in [102], supporting wired and wireless body sensor networks. It allows a wide range of hardware settings, data processing and reporting options. The proposed architecture is based on three main layers, including data acquisition, processing and communication modules. A programmable analog front-end ADAS1000 with five configurable gain single-ended channels has been implemented. In addition, a 24-bit resolution analog-to-digital converter with a programmable data rate up to 128 kHz has been designed. Moreover, three channels are provided for immediate communication and storage of results of physiological measurement in either raw or processed form.

A noise-power-area optimized biosensing front-end application specified integrated circuit (ASIC) for wireless body sensor nodes, and implantable medical devices are presented in [103]. The ASIC is implemented in a 0.18 μm CMOS process, and it is reconfigurable to accommodate different biopotentials with the high-pass and low-pass cutoff frequencies being 0.5–300 Hz and 150–10 kHz, respectively. An antialiasing filter is also available for the switching-optimized 10-b successive approximation register (SAR) analog-to-digital converter (ADC). The analog front-end (AFE) allows a programmable gain from 38 to 72 dB. Moreover, a power management unit provides the power supply, multiple reference voltages and bias currents to the entire chip. In terms of performance, the following characteristics can be identified: (i) AFE and ADC dissipate 5.74 μW and 306 nW, respectively, (ii) the measured input-referred noise is 2.98 μVrms, (iii) the noise efficiency factor is 2.6, (iv) the power efficiency factor is 9.46 and (v) the area of the AFE is 0.0228 mm^2^.

A summary of these contributions about wearable monitoring systems chosen among the papers in the literature published in the last 5 years is made in Table 2.

## 5. Commercial Wearable Devices

Wearable portable systems aim to daily acquire and processes different health data, providing early detection of pathological signs and improving the treatment and the continuous monitoring of disease. Many commercial EMG and ECG sensors are available, and they are designed and created to satisfy different specifications. In this section, the review proposes a description of the common commercial biosignal acquisition systems for physiological monitoring. These systems have been chosen to be the most used devices in health practice presenting similar characteristics to be compared.

Biometrics Ltd offers different data acquisition systems to collect analog and digital data from various sensors and are available in wireless, portable and laboratory configurations. Wireless systems furnish total freedom of movement without being constrained by wires [104]. They are available in 2-, 4-, 8- and 16-channel configurations to acquire EMG signals by using surface, small and lightweight sensors, allowing muscle activity readings to be smooth and robust with a range of up to 30 m from its receiver. The main features of these types of sensors are (i) a bandwidth from 10 Hz to 250 Hz through to 10 Hz to 5000 Hz and (ii) a sensitivity for the peak to peak measurements ranging from +/− 60 mV to +/− 6000 mV [105]. Portable systems are comprehensive packages of sensors and instrumentation for static and dynamic measurements in a clinical setting, a research centre, or at any remote location such as an office, workplace or home. Biometrics offers three different versions of EMG sensors: (i) surface EMG sensors, (ii) wireless surface EMG sensors and (iii) surface EMG amplifier.

Shimmer offers a set of individual sensors for different parameters measurement [106]. Shimmer3 EMG Unit provides a configurable digital front-end useful for the acquisition and measurement of EMG signals [107]. This unit uses a non-invasive sensor allowing registration of the activity of the whole muscle. It provides two channels of EMG data with a common reference electrode in a wireless solution. It can also acquire ECG data, but EMG and ECG data cannot be measured simultaneously from a single unit. Shimmer3 EMG unit contains an MSP430 microcontroller, a Bluetooth Radio (RN-42) and an integrated 8 GB micro SD card, and it is supplied by a 450 mAh rechargeable Li-ion battery. The Shimmer3 ECG unit is equivalent to the Shimmer3 EMG unit, but it is optimized for the measurement of physiological signals for ECG [108]. It is a four-lead ECG solution to measure bipolar limb leads chosen from V1–V6; moreover, it offers respiration demodulation from ECG data and allows lead-off detection.

BioSemi instrumentation proposes ActiveTwo biopotential measurement systems for research applications [109]. This system is characterized up to 256-channel DC amplifier, 24-bit ADC per channel and active electrodes. These active electrodes are smaller with less cable weight while offering even better specs in terms of low-frequency noise and input impedance. Specifically, the ActiveTwo system provides: (i) up to 256 + 8 electrode +7 sensor channels in a single ultra-compact box, (ii) battery-powered front-end with fiber optic data transfer, (iii) reliable measurements without skin preparation, (iv) improved digital resolution with LSB value of 31 nV and (v) user-selectable sample rate of 2, 4, 8, 16 kHz/channel. Moreover, it is suitable for EEG, ECG and EMG measurements, and it offers graphical programming in LabVIEW.

FreeEMG is an electromyography device with wireless probes for the dynamic analysis of muscle activity. It is a 4G technology device for surface EMG analysis characterized by signal accuracy, absence of wires, lightness and reduced size of the probes. FreeEMG is largely used for orthopedic and neurological disorders; pharmacological treatments; the evolution of motor deficits; rehabilitation and follow-up; and athletic task optimization. PLUX develops innovative biosignal acquisition and monitoring platforms integrating wearable body sensors such as EMG and ECG combined with wireless connectivity and software applications [110]. Two of these platforms are BITalino and Biosignalplux.

BITalino (r)evolution kit is an all-in-one board with all the blocks pre-connected and ready to work out-of-the-box [111]. This model is fitted with Bluetooth communication. Its EMG sensors are specially designed for surface EMG. The bipolar configuration is ideal for low-noise data acquisition, and the raw data output enables it to be used for human–computer interaction and biomedical projects alike.

Biosignalsplux represents an advanced wireless toolkit to collect and analyze reliable and high-definition biosignal data [112]. It offers a set of cabled and wearable sensors. The biosignalsplux electromyography (EMG) sensor is a high-performance bipolar sensor with low noise for seamless muscle data acquisition. This sensor is designed to monitor muscular activity, and the bipolar configuration is ideal for uncompromised low-noise data acquisition. The raw data output provides medical-grade data enabling it to be used for advanced and highly accurate biomedical biomechanics and sports research. Its main features are (i) bipolar differential measurement, (ii) pre-conditioned analog output, (iii) high signal-to-noise ratio and (iv) medical-grade raw data output. It is also ready-to-use, and it is miniaturized. The wireless single-channel EMG device for real-time muscle sensing is muscleBAND. It is an integrated single-channel EMG sensor with a triaxial accelerometer and magnetometer for real-time acquisition of muscle activity and motion data with an integrated dual Bluetooth module. This sensor allows data acquisitions with up to 16-bit resolution at up to 1000 Hz sampling rate, with the internal battery providing enough power for continuous data streaming.

Delsys proposes complete wireless EMG-based solutions for monitoring human movement in research, clinical and educational settings [113]. These solutions are composed of (i) research, mobile and lite systems, (ii) EMG sensors, (iii) mobile software and (iv) software for devices integration. The most used EMG sensor is Trigno Avanti Sensor, which can capture muscle activity and movement data accurately. It is designed to work with all Trigno systems, and it is characterized by (i) patented technology, (ii) improved RF performance, (iii) cable-free design, (iv) selectable EMG bandwidth settings and (v) on-board signal processing. It also allows differential EMG input acquisition in a very small dimension and weight. Trigno Research+ is a high-performing device designed to make EMG signal detection reliable and easy, offering a full set of physiological and biomechanical monitoring tools to simplify complex research and provide the highest quality data. Proprietary RF protocol guarantees synchronization between all sensors and allows data transmission from Trigno wireless sensors to a Trigno base station. Table 3 reports the main characteristics of the selected wearable monitoring systems.

## 6. Discussion

The acquisition and analysis of biopotentials (e.g., EEG, ECG and EMG) are relevant in a diagnostic and therapeutic context to monitor and identify normal and/or abnormal physical conditions. In the rehabilitation field, these biopotentials became important to support human activity monitoring. Surface EMG is a widely used technology in rehabilitation research. It furnishes quantifiable information on the myoelectric signal of a muscle. Nevertheless, there is a strong contrast between the application of sEMG in clinical practice and research findings. This contrast is due to several issues including: (i) limited time and resources due, for example, to electrodes and skin preparations, electrode placements and equipment setup; (ii) clinically inapplicable sEMG system features mainly caused by the limited spatial resolution of sEMG; and (iii) lack of training and confidence in utilization of sEMG technology. Common example of issues are technical and regard signal processing and information extraction algorithms which do not directly produce clinically relevant information. Other issues concerns the user-unfriendliness of some equipment. Finally, the cost of the devices and the timing procedure to perform a measurement and to obtain a clinically useful information have also a negative impact. In terms of sensor technology, the main characteristics of a wearable system include low-energy operations, light weight and safety requirements. In addition, a wearable device must be compact and comfortable enough to be easily used. Moreover, it must be portable and guarantee continuous monitoring of human behavior through a battery power supply and wireless connectivity, respectively. Today, many commercial sensors are available to meet these requirements, and they offer different personalized solutions regarding specific needs. These sensors are characterized by small dimensions and portable configurations, making them more useful in patient control in clinical structure and at home. The limitation of modern commercial sensors is mainly due to the high cost and the complexity of the system used by a single subject. Moreover, they are not always easy to use. One of the main problems of existing biosignal technology is the high cost of commercial devices that is higher in devices with wireless technologies, which makes it easier to install on the muscle and connect with the computer; however, this requires a more complex system configuration. Very often, these commercial sensors present complex design, mainly based on sophisticated hardware with little attention toward a comfortable sensor–patient interface. Another problem regards the fact that these sensors are designed based on bench tests, models and patient simulators, ignoring the key component and working well in the simulation phase but revealing problems under real-life conditions. To improve these limitations, research prototypes are becoming very important because the newest sensor technology must meet the needs of clinicians and patients. The new generations of sensor-driven technologies should individuate the potential clinical need, identifying the key biosignals related to a specific physiological processes and seeking the development of platforms based on novel sensing technologies. These new platforms must be able to monitor the physiological processes from useful locations using novel transduction mechanisms. The hardware also should be designed and developed around these constraints and requirements. In the future, these sensors will support home-based physiological data collection, preventive healthcare programs and also facilitate remote care and rehabilitation protocols. For this reason, the future steps will regard the development of miniaturized and economic devices which will be used on a large population.

## 7. Conclusions

This contribution is proposed as a review of the most common physiological monitoring systems in the rehabilitation field. It focuses on the EMG signal front-end as one of the main platforms to support physicians, patients and general subjects in rehabilitation protocols. In this review, many references in the literature have been presented to highlight the importance of the acquisition, analysis and monitoring of EMG signals to muscular activity control. Moreover, different commercial available EMG sensors have also been described and compared to identify the most common features of these devices in EMG acquisition. The main characteristics are portability and wireless data transfer, aiming to ensure a practical use in rehabilitation.

## Figures and Tables

**Table 1:** Databases used for this review.

Database	URL	Date Access
PubMed	https://pubmed.ncbi.nlm.nih.gov/	30 June 2021
MDPI	https://www.mdpi.com/	30 June 2021
Springer	https://link.springer.com/	30 June 2021
ACM Digital Library	https://dl.acm.org/	30 June 2021
Science Direct	https://www.sciencedirect.com/	30 June 2021

**Table 2:** Summary of selected wearable monitoring system included in this review.

Authors	Signals	Channels	Platform Characteristics	Features
Tran et al., 2021	Bio-potentials	4 channels	Four-channel neural recording analog front-end composed by a low-noise amplifier (LNA), a programmable gain amplifier (PGA) and buffers; 4-to-1 multiplexer (MUX) and analog-to-digital converter (ADC)	Programmable gain from 45 dB to 63 dB, input-referred noise of 3.16 μVRMS within the 10 kHz bandwidth, noise efficiency factor of 2.04, power efficiency factor of 4.16, power consumption of 2.82 μW per channel powered from 1 V supply voltage
Yin et al., 2021	Bio-potentials, impedance respiration	Single 1 channel	Oversampling and fast digital lock-in technology, ADS1294R, STM32F103RET6 for signal processing	Improve the common-mode rejection ratio (CMRR) and the signal-to-noise ratio (SNR) of the signal
Zhao et al., 2020	ECG/EMG	N.A.	Low-energy Bluetooth module	Wearable monitoring device, software platform for data analysis
Biagetti et al., 2020	Bio-potentials	3 channels	Six electrodes, 24 bits of resolution and a sampling rate up to 3.2 kHz for each channel, Bluetooth Low Energy wireless link	Wireless sensor, real-time acquisition, maximization of the available bandwidth, reliability of the transmission
Nakamura et al., 2020	ECG/EMG	N.A.	Analog front-end (AFE)	Capacitive measurements
Liu et al., 2019	Bio-potentials	8 channels	Powerful microcontroller unit, lithium battery, Bluetooth 3.0 data transmission and built-in 2 GB flash memory	Portable device with a graphic user interface (GUI) and an application program for displaying the signals on a computer or a smart device
Park et al., 2018	Bio-potentials	128 channels	Energy-efficient integrated circuit architecture of a Δ-modulated ΔΣ AFE with multi-shank neural probes connected to individual AFEs	The Δ-ΔΣ AFE is characterized by a consume of each single-channel AFE of 3.05 μW from 0.5 and 1.0 V supplies in an area of 0.05 mm^2^ with 63.8 dB signal-to-noise-and-distortion ratio and 3.02 noise efficiency factor
Raheem et al., 2018	Bio-potentials	2 channels	Programmable gain amplifier (PGA) and 10-bitΣΔ (SDM-ADC)	High impedance, power consumption of 11 mW, programmable gains from 52.6 dB to 72 dB and input referred noise of 3.5 µV in the amplifier bandwidth
Mazzetta et al., 2018	EMG	Differential 1 channel	32 bit ARM® Cortex®-M4, microSD, Bluetooth 4.0, 592 mWh battery, micro-USB connector, 30 × 30 × 15 mm dimensions, weight of 10 g	Power consumption, compactness and energy autonomy, wireless and comfortably wearable
Biagetti et al., 2018	sEMG	N.A.	Ultralight wireless sensing nodes, base station for data transmission through a 2.4 GHz radio link, communication protocol designed on top of the IEEE 802.15.4 physical layer	Low-cost wearable wireless system, user interface software for viewing, recording and analyzing data
Kast et al., 2017	Bio-potentials	Bipolar 64 channels	Up to eight front-end acquisition modules with synchronization module, a separated universal serial bus data-link to the computer and an ADS1299	Raw data are analyzed and stored on a personal computer or a single-board computer
Sarker et al., 2017	ECG/EMG	8 channels	24 bit resolution/channel and 500 samples/s, IoT-based system	Compact and wearable portable bio-signal acquisition device, real-time data wireless transmission, low energy consumption
Li et al., 2017	ECG/EMG	N.A.	150 mAh rechargeable Li-ion battery, packaged into a 39 × 32 × 17 mm 3D printed small box, total weight of 24.0 g, power management circuit, dual power supply for operational amplifiers	Wearable wireless non-contact system, ultra-high input impedance, feasibility of long-term biopotential monitoring
Senepati et al., 2017	ECG/EMG	N.A.	Band pass and band stop FIR filters, Successive Approximation Register (SAR) DAC, Spartan-3E FPGA and 0.18 μm CMOS TSMC technology	Area of 33,005 μm^2^ area, power consumption of 0.382 mW, suppressing of baselines wander and power line interference noise (50/60 Hz)
Bhamra et al., 2017	ECG/EMG	N.A.	ASIC technology in a 0.18 μm CMOS process, high-pass and low-pass cutoff frequencies being 0.5–300 Hz and 150 Hz–10 kHz, antialiasing filter, successive approximation register (SAR) analog-to-digital converter (ADC), power management	Wireless, programmable gain from 38 to 72 dB, AFE and ADC dissipation of 5.74 μW and 306 nW, measured input-referred noise of 2.98 μVrms, noise efficiency factor of 2.6, power efficiency factor of 9.46, area of the AFE of 0.0228 mm^2^
Kim et al., 2016	Bio-potentials, PPG, BIA	N.A.	CMOS technology, low-power and multimodal analog front-end (AFE)	Wearable health monitoring, low dimension and power consumption
Mahmud et al., 2016	ECG	N.A.	Fully integrated analog front-end (AFE), temperature sensor, accelerometer, Bluetooth Low Energy (BLE) module	Multiparameter real time monitoring, small dimensions, Android application, alerts
Piccinini et al., 2016	ECG/EMG	N.A.	ADS1294 Medical Analog Front End, CC3200 microcontroller, two Li-ion charged batteries	Portable solution, size physical reduction, robustness in wireless transmission, reliability in data acquisition and processing
Lee et al., 2016	ECG/EMG	N.A.	Mixed-signal processor system-on-chip (SoC), Bluetooth Low Energy (BLE) chip, 200 mAh battery	Wireless transmission, power efficiency, 12 h of continuous recording
Augustyniak et al., 2016	Bio-potentials	Single-ended 5 channels	Programmable AFE ADAS1000, 24-bit resolution analog-to-digital converter with programmable data rate up to 128 kHz	Wired and wireless body sensor networks, configurable gain for channel

**Table 3:** Main characteristics of the commercial wearable monitoring systems.

Features	Biometric	Shimmer	Biosemi	BTS Bioengineering	Biosignal Plux	BITalino	Delsys
Type of sensor	Wireless EMG Sensor	Shimmer3 EMG Unit	ActiveTwo	FreeEMG 1000 H_2_O	Electro-myography Sensor	Electro-myography Sensor	Trigno Avanti Sensor
Size (mm × mm × mm)	42 × 24 × 14	65 × 32 × 12	120 × 150 × 190	Probes: 41.5 × 24.8 × 14	28 × 70 × 12	12 × 27	27 × 37 × 13
Weight	17 g	31 g	1.1 kg	13 g—battery included	25 g	N.A.	14 g
# channels	1	2	8 up to 256	1	1	1	1 differential input
Input impedance	>100 Mohms	N.A.	>100 M @ 50 Hz		>100 GOhm	10/7.5 GOhm/pF	
Input range	+/−6 mV	Approx. 800 mV @ gain = 6	+262 mV to −262 mV	N.A.	Up to 10 mV	±1.64 mV @ VCC = 3.3 V	11 mV/22 mV rti
Gain	+/−60 mV to +/−6000 mV	1,2,3,4,6,8,12 (software configurable)	N.A.	N.A.	1000	1009	11 mV/22 mV rti
CMRR	>96 dB (typically 110 dB) @ 60 Hz	N.A.	>90 dB @ 50 Hz	N.A.	100 dB	86 dB	<−80 dB
Consumption	N.A.	N.A.	4 Watt @ 280 channels	N.A.	1 mA	0.17 mA	N.A.
Bandwith	0–250, 470, 950, 5000 Hz	8.4 kHz	Up to DC—3200 Hz @ –3 dB	N.A.	25–500 Hz	25–482 Hz	10–850 Hz 20–450 Hz
Data transmission	Wireless	Bluetooth Radio – RN-42	Fiber optic	Wireless IEEE 802.15.4	Bluetooth Low Energy	N.A.	2.400-2.483 GHz ISM Band, Proprietary RF Protocol - BLE V4.2
Resolution	N.A.	24 bit	24 bit	16 bit	12 bit	N.A.	16 bit
Sample rate	N.A.	125, 250, 500, 1000, 2000, 4000, 8000 SPS	2048 Hz–4096 Hz–8192 Hz–16,384 Hz	N.A.	N.A.	N.A.	4370 sa/sec
Battery type and life	Rechargeable Li-ion Polymer, Up to 8 h	450 mAh rechargeable Li-ion battery	Battery power with >10 h @ 144 channels, >72 h @ 16 channels	Battery Li-Po, Up to 6 h	N.A.	Battery Li-Po 700 mAh	Rechargeable Li-Po Battery Up to 8 h

## Abbreviations

The following abbreviations are used in this manuscript:

EEG	Electroencephalogram
ECG	Electrocardiogram
EMG	Electromyogram
sEMG	Surface Electromyography
IC	Integrated Circuit
NMES	Neuromuscular Electrical Stimulation
BCI	Brain–Computer Interface
ADC	Analog-to-Digital Converter
CMRR	Common-Mode Rejection Ratio
SNR	Signal-to-Noise Ratio
AFE	Analog Front-End
PGA	Programmable Gain Amplifier
LNA	Low-Noise Amplifier
BLE	Bluetooth Low Energy
BIA	Bioelectrical Impedence Analyzer
CBM	Capacitive Biopotential Measurements
SoC	System-on-Chip
ASIC	Application Specified Integrated Circuit
